# Geographic Variation of *Phyllodiaptomus tunguidus* Mitogenomes: Genetic Differentiation and Phylogeny

**DOI:** 10.3389/fgene.2021.711992

**Published:** 2021-08-31

**Authors:** Xiao-Li Zhang, Ping Liu, Shao-Lin Xu, Eric Zeus Rizo, Qun Zhang, Henri J. Dumont, Bo-Ping Han

**Affiliations:** ^1^Department of Ecology, Jinan University, Guangzhou, China; ^2^Division of Biological Sciences, College of Arts and Sciences, University of the Philippines Visayas, Iloilo, Philippines; ^3^Department of Biology, Ghent University, Ghent, Belgium

**Keywords:** tRNA-Arg, calanoid, southern China, genetic differentiation, phylogeny

## Abstract

*Phyllodiaptomus tunguidus* (Copepoda: Calanoida) is largely endemic to and widespread in freshwater in southern China, where it inhabits a complex landscape from lowland to highland across an elevation gradient of 2000m. A deep genetic differentiation can be expected between its most distant geographic populations. Here, we sequenced nine mitogenomes from diverse populations. All mitogenomes contained 37 genes, including 13 protein-coding genes (PCG), two rRNA genes, 22 tRNA genes and one control region. Their base composition, genetic distance and tRNA structure indeed revealed a wide differentiation between mitogenomes. Two *P. tunguidus* from Guangxi near Vietnam differed from the other seven by up to 10.1%. Their tRNA-Arg had a complete clover-leaf structure, whereas that of the others did not contain an entire dihydrouridine arm. The nine mitogenomes also differed in the length of rRNA. NJ, ML, and Bayesian analyses all split them into two clades, *viz.* the two *P. tunguidus* from Guangxi (Clade 1), and the other seven (Clade 2). Both the structure and phylogeny of the mitogenomes suggest that *P. tunguidus* has complex geographic origin, and its populations in Clade 1 have long lived in isolation from those in Clade 2. They currently reach the level of subspecies or cryptic species. An extensive phylogenetic analysis of Copepoda further verified that Diaptomidae is the most recently diverging family in Calanoida and that *P. tunguidus* is at the evolutionary apex of the family.

## Introduction

Copepods are omnipresent in aquatic ecosystems. They are extremely abundant in freshwater and constitute a major component of most planktonic, benthic and groundwater communities, including semi-terrestrial situations such as damp mosses and leaf litter on forest floors (Boxshall and Jaume, 2000; Boxshall and Defaye, 2008). Of an estimated 13,000 morphospecies known, about 2,800 species inhabit freshwater. Freshwater copepods are comprised of five orders (Calanoida, Harpacticoida, Cyclopoida, Gelyelloida, and Siphonostomatoida; Boxshall and Halsey, 2004). Among them, calanoids show interesting ecological characteristics (Andronov, 1974; Park, 1986; Blanco-Bercial et al., 2011). Diaptomidae (Sars, 1903) is the dominant family in inland waters of Europe, Asia, North America, Africa, and northern low-altitude South America.

Most freshwater Diaptomidae are planktonic, inhabiting the pelagic (Boxshall and Defaye, 2008). They are filter-feeding and an important regulator of bottom-up (available resources) and top-down (consumers) effects in freshwater zooplankton communities (Gliwicz, 2002; Lin et al., 2018). They respond quickly to environmental changes and most are endemic to well-defined regions (Matsumura-Tundisi and Tundisi, 2003; Boxshall and Defaye, 2008). As copepods have a high dispersal potential, it is interesting to know how much their populations differentiate within species’ ranges (Marrone et al., 2013). Molecular phylogenetic analysis with mitochondrial DNA has been used in a variety of studies including evolutionary genomics, systematics, and molecular evolution has become a robust and reliable tool due to such unique features of mitogenomes as high mutation rates, an absence of introns, and maternal inheritance (Ki et al., 2009). In calanoids, several partial mitochondrial sequences, such as *Cytb*, *COI*, and *16S rRNA*, have been utilized for species diagnoses (Marrone et al., 2013; Perbiche-Neves et al., 2015; Previšić et al., 2016), phylogenetic analysis (Blanco-Bercial et al., 2011) and population genetics (Makino and Tanabe, 2009; Chen and Hares, 2011). For a phylogenetic relationship multiple gene analysis is more powerful than analysis using single markers (Blanco-Bercial et al., 2011). Complete mitochondrial sequences can provide improved resolution and sensitivity in such investigations. The complete mitogenomes of copepods are typically circular molecules, approximately 16kb in length (Jooste et al., 2019). Overall, they consist of 37 genes, including 13 protein coding genes (PCGs), two ribosomal RNA genes (rRNAs), 22 transfer RNA genes (tRNAs), and a control region.

To date, only two complete mitochondrial genomes have been isolated from Diaptomidae, a tiny fraction of the number of known species (Jooste et al., 2019; Zhang et al., 2020b). Here, we discuss another eight geographical mitogenomes of *Phyllodiaptomus tunguidus*, a freshwater species, widespread in a variety of waters including ponds, lakes and reservoirs in southern China (Chen, 1990; Dumont and Reddy, 1993; Xue et al., 2006). Shen and Tai (1964) first described it from Guangdong Province, southern China. Dumont and Reddy (1993) re-described the species from the River Li in Guangxi and Lake Erh in Yunnan Province, also southern China, and they characterized it as a Chinese endemic (Dumont and Reddy, 1993). Literature and our own field investigation show that most of *P. tunguidus* is currently distributed in the Pearl River Basin, but also in Hunan, Sichuan and Fujian provinces (Chen, 1990; Dumont and Reddy, 1993). From lowland (Guangdong) to highland (Yunnan), this includes a complex landscape with an elevation gradient of 2000m, mainly pertaining to the Pearl River Basin. This large area not only has a strong environmental gradient, but also a complex geological history, affected by the uplift of the Tibetan Plateau (Hall, 2002; Clark et al., 2004; Zheng, 2015). Therefore, a deep genetic differentiation is expected between at least the most extreme of its geographic populations. To document this, we here sequenced eight mitogenomes of far-apart geographical origin plus the mitogenome we previously sequenced (Zhang et al., 2020b). We searched for differences in genomic structure, base composition, codon usage, gene order, and other genetic information. We also conducted an extensive phylogenetic analysis that included 11 species from other copepod groups. Such a comparative analysis of whole mitogenomes was expected to provide new insights into the evolutionary relationships in Copepoda and to reaffirm the taxonomic position of *P. tunguidus*.

## Materials and Methods

### Sample Collections, Identification, and DNA Extraction

**Figure 1:**
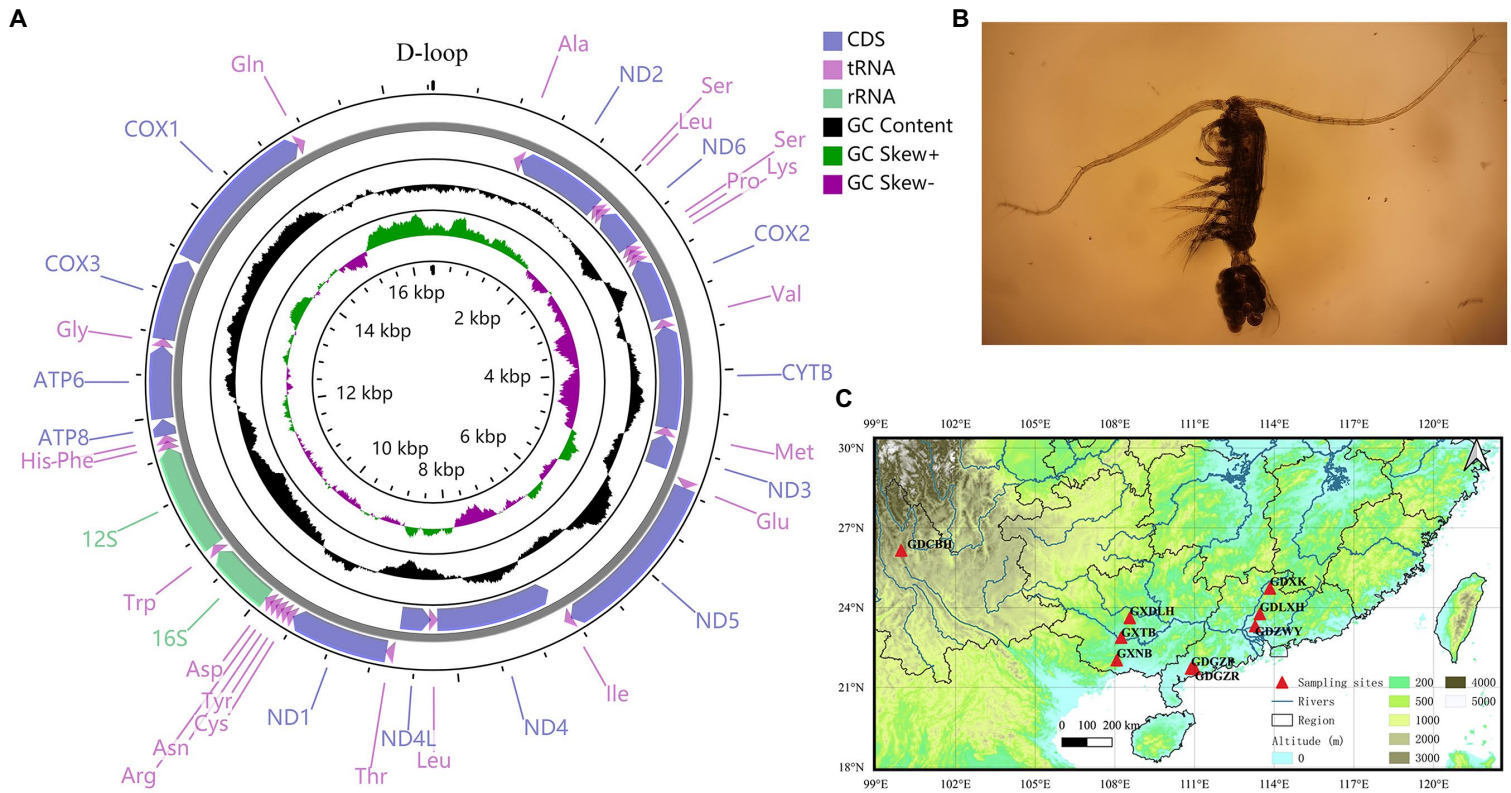
**(A)** Circular map of the mitochondrial genome of *Phyllodiaptomus tunguidus*. **(B)** *P. tunguidus* female **(C)** sample localities (*N*=9) in southern China.

Eight populations of *P. tunguidus* were sampled across southern China, one from Yunnan Province [Cibi Lake in Dali (CBH, 99.95°E, 26.15°N)], three from Guangxi Province, [Naban Reservoir (NB, 108.07°E, 22.02°N), Tianbao Reservoir (TB, 108.24°E, 22.87°N), Dalonghu Reservoir (DLH, 108.56°E, 23.61°N)], and four from Guangdong Province [Gaozhou Reservoir (GZR, 111.01°E, 21.69°N), a pond in Gaozhou (GZP, 110.87°E, 21.71°N), Xiaokeng Reservoir (XK, 113.84°E, 24.72°N) and a pond in Guangzhou Botanical Garden (ZWY, 113.283°E, 23.31°N; Figure 1)]. One previously sequenced mitogenome (GenBank accession: MN927223) came from Liuxihe Reservoir (LXH, 113.46°E, 23.76°N) in Guangdong Province, also southern China. The samples were fixed in 100% ethanol for total DNA extraction. All the specimens were identified following the description and illustration in Dumont and Reddy (1993). Total genomic DNA was extracted by the TIANamp Marine Animals DNAKit (TIANGEN BIOTECH CO., LTD) according to the manufacturer’s instructions. In order to verify whether these eight specimens represent the entire distribution range, we constructed a phylogenetic tree based on *Cytb* sequences (details in the Supplementary Table S2). The Bayesian tree of *Cytb* built with BEAST split *P. tunguidus* into two Clades with strong support (Supplementary Figure S1).

### Mitogenome Sequencing, Assembly, and Annotation

We sequenced and assembled the complete mitochondrial genomes of *P. tunguidus* following the next-generation method. A sequence library was generated from the genomic DNA using an Illumina Hiseq 2,500 platform. COI sequence (GenBank accession: MN12844) was used as reference seed for assembly by MITObim v.1.8 (Hahn et al., 2013; Xu et al., 2017). The contigs were extended using the assembly parameters with minimum overlap of 50bp and minimum overlap similarity of 95%.

The mitochondrial genomes were annotated with MITOS WebServer (Bernt et al., 2013) and verified *via* BLAST (Altschul et al., 1990). Transfer RNA genes were double-checked with tRNAscan-SE v.2 (Love and Eddy, 1997) and ARWEN v.1.2.3 (Laslett and Canback, 2008). Secondary structures of tRNA were predicted by tRNAscan-SE. PCGs were identified by searching open reading frames using BLAST searches in NCBI^1^ with invertebrate mitochondrial genetic codes. The ribosomal RNA genes (rRNAs) were identified by the boundary of the tRNA genes and by BLAST searches in NCBI. Non-coding regions were identified based on their sequence homology using Clustal X (Thompson et al., 1997) and MAFFT 7.2 (Katoh and Standley, 2013). The gene map of *P. tunguidus* was generated using CG View (Stothard and Wishart, 2005). The nine mitogenomes were grouped into four groups according to similarity in gene characteristic: (1) GZR, GZP, and CBH; (2) LXH, ZWY, and XK; (3) NB and TB; and (4) DLH. One representative mitogenome from each group, i.e., four mitogenome were selected for detailed analysis, the information of the other mitogenomes is shown in the supplements. The base composition and the relative synonymous codon usage (RSCU) were obtained using MEGA X (Kumar et al., 2018). The K2P genetic distances between PCGs were also calculated in MEGA X (Kumar et al., 2018). The rate of non-synonymous substitutions (Ka), rate of synonymous substitutions (Ks), and ratio of Ka/Ks were determined by DnaSP 5.1 (Librado and Rozas, 2009). Strand asymmetry was calculated using the formula: AT-skew=(A-T)/(A+T); GC-skew=(G-C)/(G+C; Perna and Kocher, 1995).

### Genetic Differentiation and Phylogenetic Analysis

To estimate the pairwise genetic distance in MEGA X (Kumar et al., 2018), all mitogenomes were pooled. A mantel test was applied to two matrixes of pairwise genetic distance and geographical distance to detect possible patterns of isolation by distance (IBD) using the R package ade4 (Dray and Dufour, 2017). A geographic distance matrix was obtained using the function “distm” in R packages “geosphere.” The distance between the sampling sites was calculated with the “Vincenty’s formula (ellipsoid)” method (Vincenty, 2013).

To verify the taxonomic location of *P. tunguidus* in copepoda, we carried out phylogenetic analysis based on an alignment of the nucleotide sequences of 13 PCGs. We obtained the concatenated nucleotide sequences of 13 PCGs from another 11 Copepoda species downloaded from GenBank for further analysis (Supplementary Table S1). The 11 species included five Calanoida species (one Diaptomidae, one Temoridae, and three Calanidae species), two Cyclopoida (one Lernaeidae and one Cyclopettidae species) and four Harpacticoida species (one Miraciidae and three Harpacticidae). We used *Diaphanosoma dubium* (*Sididae*, Ctenopoda, Branchipoda) as outgroup (GeneBank accession numbers: MG428405.1). Sequences of 13 PCGs and two rRNAs were used for phylogenetic analysis. PCGs were aligned based on amino acid sequences and rRNAs were aligned based on nucleotide sequences with MEGA X (Kumar et al., 2018). A Monte Carlo Markov Chain (MCMC) based Bayesian consensus tree was constructed using BEAST 2.4.3 (Bouckaert et al., 2014). Maximum Likelihood (ML) tree and Neighbor (NJ) tree were constructed using MEGA X (Kumar et al., 2018). The best fit model of substitution and best partition schemes for the dataset were identified with the corrected Akaike information criterion (AICc; Akaike, 1974) using PhyloSuite 1.1.15 (Zhang et al., 2020a). GTR+G+I, was chosen as the optimal model of the evolution of 13 PCGs. We performed Bayesian inference analysis using MCMC chains from 100 million generations and sampled one tree at every 1,000 generations using a burn-in of 5,000 generations. ML and NJ analyses were calculated with 1,000 bootstrap replications. The resulting phylogenetic trees were visualized in FigTree v1.4.0.

## Results

### Sequence Characteristics of Complete Mitogenome of *P. tunguidus*

We obtained eight mitochondrial genomes of *P. tunguidus* and submitted them to GenBank (Accession no: MW971441-MW971448). Including the one we published previously (Accession no: MN927223), all nine mitogenomes contained 13 PCGs, 2 rRNA (12S rRNA and 16S rRNA), 22 tRNA, and one control region (Figure 1; Table 1; Supplementary Table S3). The size and genetic sequencing of the nine *P. tunguidus* were similar, and the 37 genes were uniformly distributed in the H and L strands. Twenty-one genes are on the H-strand (13 tRNAs, seven PCGs, and two rRNAs), while 16 genes are on the L-strand (nine tRNAs and seven PCGs). All PCGs initiate strictly with ATA, ATT and ATG as the start codon. The PCGs end up strictly with TAA and TAG stop codons. The nine mitochondrial control regions were all flanked by tRNA-Gln and tRNA-Ala.

**Table 1:** Annotation of four representative complete mitochondrial genomes of *P. tunguidus*.

Gene	DLH		NB		GZR		LXH		Size	Strand	Codon		Anticodon
	From	To	From	To	From	To	From	To			Start	Stop	
tRNA ^Ala^	1	60	1	60	1	60	1	60	60	L			tgc/tgc/tgc/tgc
ND2	59	1,027	59	1,027	59	1,027	59	1,027	969	L	ATA/ATA/ATA/ATA	TAG/TAG/TAG/TAG	
tRNA^Ser^	1,028	1,083	1,028	1,083	1,028	1,083	1,028	1,083	56	L			tga/tga/tga/tga
tRNA ^Leu^	1,082	1,143	1,082	1,143	1,082	1,143	1,082	1,143	62	L			gtc/gtc/gtc/gtc
ND6	1,142	1,603	1,141	1,605	1,142	1,603	1,142	1,603	462/465/462/462	L	ATT/ATT/ATT/ATT	TAA/TAA/TAA/TAA	
tRNA ^Ser^	1,603	1,660	1,603	1,660	1,603	1,660	1,603	1,660	58	L			tct/tct/tct/tct
tRNA ^Pro^	1,660	1721	1,660	1721	1,660	1721	1,660	1721	62	L			tcc/tcc/tcc/tcc
tRNA^Lys^	1723	1783	1723	1783	1723	1783	1723	1783	61	L			gaa/gaa/gaa/gaa
COX2	1783	2,484	1783	2,484	1783	2,484	1783	2,484	702	L	ATT/ATT/ATT/ATT	TAA/TAA/TAA/TAA	
tRNA ^Val^	2,503	2,562	2,503	2,562	2,503	2,562	2,503	2,562	60	L			gtg/gtg/gtg/gtg
CYTB	2,562	3,695	2,562	3,695	2,562	3,695	2,562	3,695	1,134	L	ATG/ATG/ATG/ATG	TAA/TAA/TAA/TAA	
tRNA ^Met^	3,698	3,759	3,698	3,759	3,698	3,759	3,698	3,759	62	L			gta/gta/gta/gta
ND3	3,767	4,120	3,767	4,120	3,767	4,120	3,767	4,120	354	L	ATT/ATG/ATT/ATT	TAA/TAA/TAA/TAA	
tRNA ^Glu^	4,162	4,226	4,160	4,223	4,162	4,226	4,162	4,226	65	H			ttc/ttc/ttc/ttc
ND5	4,227	5,912	4,224	5,909	4,227	5,912	4,227	5,912	1,686	H	ATA/ATA/ATA/ATA	TAA/TAA/TAA/TAA	
tRNA ^Ile^	5,911	5,972	5,908	5,969	5,911	5,972	5,911	5,972	62	H			gat/gat/gat/gat
ND4	5,970	7,250	5,967	7,247	5,970	7,250	5,970	7,250	1,281	L	ATA/ATA/ATA/ATA	TAA/TAA/TAA/TAA	
tRNA^Leu^	7,260	7,323	7,257	7,320	7,260	7,323	7,260	7,323	64	L			taa/taa/taa/taa
ND4L	7,319	7,648	7,316	7,645	7,319	7,648	7,319	7,648	330	L	ATT/ATT/ATT/ATT	TAA/TAA/TAA/TAA	
tRNA ^Thr^	7,686	7,746	7,684	7,744	7,686	7,746	7,686	7,746	61	H			gca/gca/gca/gca
ND1	7,791	8,711	7,790	8,710	7,791	8,711	7,791	8,711	921	H	ATA/ATA/ATA/ATA	TAG/TAG/TAG/TAG	
tRNA ^Cys^	8,711	8,771	8,710	8,770	8,711	8,771	8,711	8,771	61	H			tgt/tgt/tgt/tgt
tRNA ^Tyr^	8,772	8,833	8,771	8,832	8,772	8,833	8,772	8,833	62	H			cat/cat/cat/cat
tRNA ^Asn^	8,836	8,895	8,835	8,895	8,836	8,895	8,836	8,895	60/61/60/60	H			gtt/gtt/gtt/gtt
tRNA ^Arg^	8,899	8,956	8,898	8,955	8,899	8,956	8,899	8,956	58	H			tcg/tcg/tcg/tcg
tRNA ^Asp^	8,957	9,019	8,956	9,018	8,957	9,019	8,957	9,019	63	H			tag/tag/tag/tag
rrnS	9,018	9,660	9,017	9,657	9,018	9,660	9,018	9,660	643/641/643/643	H			
tRNA ^Trp^	9,666	9,733	9,663	9,730	9,666	9,733	9,666	9,733	68	H			tca/tca/tca/tca
rrnL	9,734	10,776	9,730	10,773	9,734	10,772	9,734	10,773	1043/1044/1039/1040	H			
tRNA ^Phe^	10,776	10,835	10,773	10,832	10,772	10,831	10,773	10,832	60	H			ttt/ttt/ttt/ttt
tRNA ^His^	10,834	10,895	10,831	10,892	10,830	10,891	10,831	10,892	62	H			tac/tactac/tac
ATP8	10,896	11,057	10,893	11,054	10,892	11,053	10,893	11,054	162	H	ATT/ATT/ATT/ATT	TAA/TAA/TAA/TAA	
ATP6	11,060	11,761	11,057	11,758	11,056	11,757	11,057	11,758	702	H	ATG/ATG/ATG/ATG	TAG/TAA/TAG/TAG	
tRNA ^Gly^	11,765	11,820	11,762	11,817	11,761	11,816	11,759	11,822	56/56/56/64	H			tgg/tgg/tgg/tgg
COX3	11,822	12,613	11,819	12,610	11,818	12,609	11,819	12,610	792	H	ATG/ATG/ATG/ATG	TAA/TAA/TAA/TAA	
COX1	12,633	14,168	12,631	14,166	12,629	14,164	12,630	14,165	1,536	H	ATT/ATT/ATT/ATT	TAA/TAA/TAA/TAA	
tRNA^Gln^	14,169	14,234	14,167	14,232	14,165	14,230	14,166	14,231	66	H			ttg/ttg/ttg/ttg
D-Loop	14,236	-	14,233	-	14,231	-	14,232	-	-				

### Comparative Analysis of Nine Mitogenomes of *P. tunguidus*

#### Base Composition

The nucleotide composition of the nine mitogenomes showed A+T bias (whole genomes AT contents: 68.83–69.49%; PCGs: 66.43–66.9%; Table 2; Supplementary Table S4). The AT-skew is negative for both whole genomes (−0.002 to −0.013) and 13 PCGs (−0.227 to −0.234), i.e., a higher occurrence of Ts than As. In addition, the first, the second and the third codon of PCGs showed a different base composition (especially A+T content and G content) and bias against T. The A+T content of PCGs at the 3rd codon position is higher than that of 1st and 2nd codon positions, and the G content at the 3rd codon position was lower than that 1st and 2nd codon positions. Bias against T is prominent in the whole genome (34.8–34.9%), all protein-coding genes (PCGs; 41.0–41.2%), the first (35%), the second (47%) and the third (41–42%) codon of PCGs, especially at the second codon.

**Table 2:** A+T contents, AT-skew of the four representative mitogenomes of *P. tunguidus*.

	Location	Whole genome	Protein-coding genes	First codon position	Second codon position	Third codon position	rRNAs
A+T contents	DLH	69.18	66.82	62.93	63.96	73.56	77.28
	NB	68.83	66.43	62.44	64.07	72.77	76.78
	GZR	69.49	66.86	62.74	63.96	73.86	77.29
	LXH	69.46	66.90	62.75	64.08	73.86	77.48
AT-skew	DLH	−0.007	−0.233	−0.107	−0.469	−0.136	−0.001
	NB	−0.013	−0.234	−0.111	−0.473	−0.134	−0.015
	GZR	−0.003	−0.227	−0.107	−0.469	−0.118	0.005
	LXH	−0.002	−0.228	−0.108	−0.469	−0.122	0.005

#### Protein-Coding Genes and Codon Usage

There were differences in PCGs among nine *P. tunguidus*. ND3 started with ATG in two mitogenomes collected at NB and TB from Guangxi Province (near Vietnam), but with ATT in the other seven mitogenomes. The ATP6 gene stopped with TAA in the two *P. tunguidus* at NB and TB but with TAG in the other seven *P. tunguidus*. This difference indicated that the *P. tunguidus* from NB and TB in Guangxi Province and those from the other seven sites might have undergone selection for different mechanisms of transcriptional initiation and termination during evolution. Gene-overlap was detected between tRNA and tRNA and between tRNA and PCG, but not between PCG and PCG. tRNA-Ser and tRNA-Leu overlapped by two nucleotides and tRNA-Leu – ND4L shared five nucleotides.

**Figure 2:**
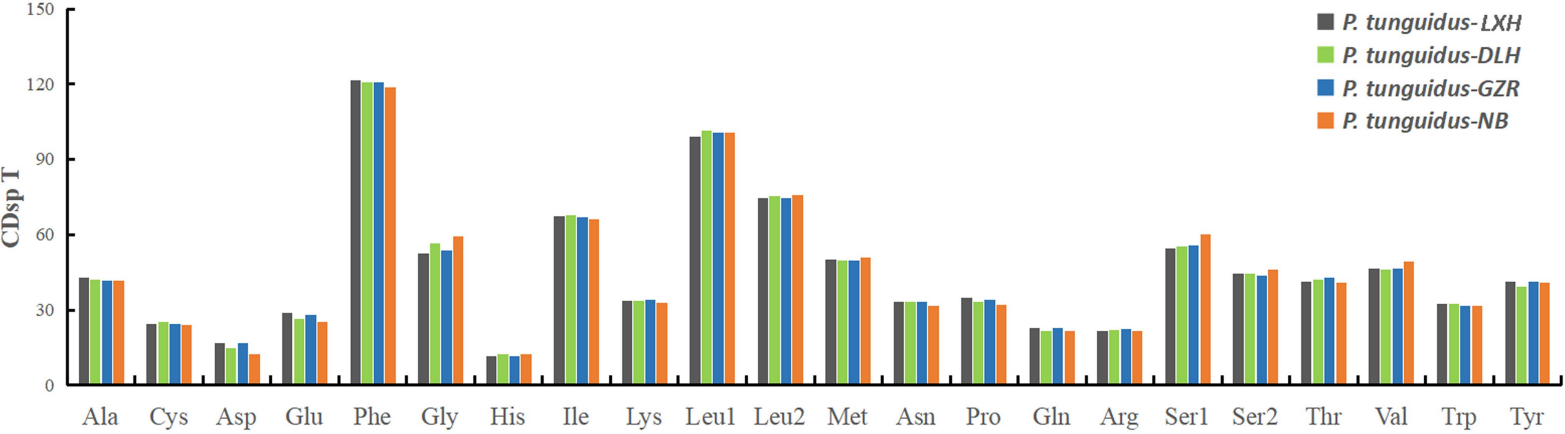
The codon usage pattern for the four representative mitogenomes of *P. tunguidus*. The codon families are shown on the *X*-axis and CDsp T on the *Y*-axis.

The total length of the PCGs was 11, 034bp for the two *P. tunguidus* from TB and NB and 11, 031bp for the others. Excluding stop codons, the 13 PCGs in the nine mitogenomes consisted of 3, 664–3, 665 codons (CDs) in total and showed similar properties in terms of codon usage (Figure 2). The two most predominant codon families were phenylalanine (Phe), and leucine (Leu1, UUR), each with more than 100 CDsp T (codons per thousand codons). Among them, Phe exhibited the highest usage bias (118.9–121.5 CDsp T), which might be associated with the coding function of the chondriosome. In contrast, His (11.55–12.40 CDsp T) showed the smallest number of CDsp T.

**Figure 3:**
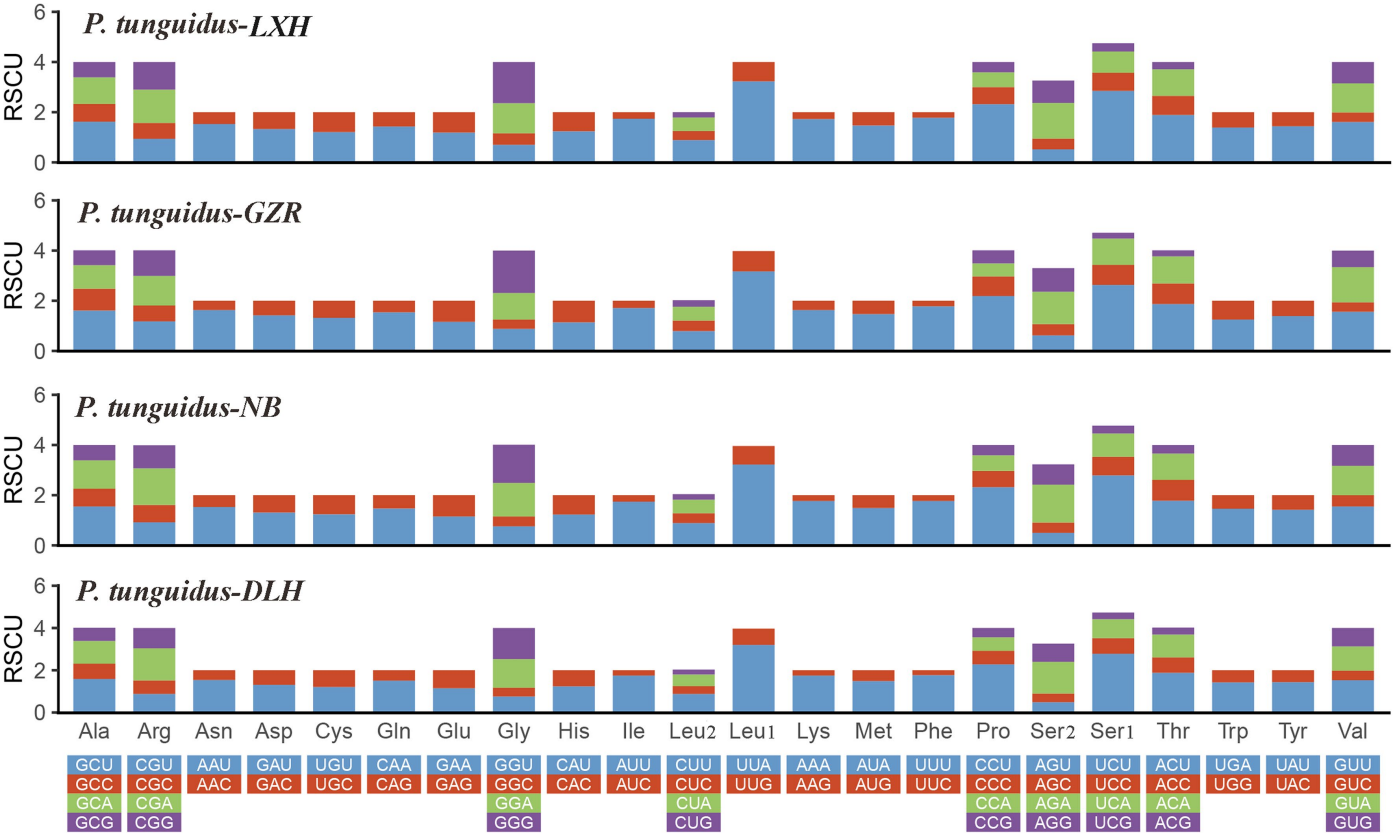
Relative synonymous codon usage in the four representative mitogenomes of *P. tunguidus*. Codon families are indicated below the *X*-axis.

Subsequently, we evaluated the relative synonymous codon usage (RSCU) to determine the preference for specific synonymous codons. The codon usage is generally similar among the four representative mitogenomes. The high A+T content in the frequently used codons (i.e., UUA, CCU, UCU; Figure 3) effectively contributes to the high A+T composition in the PCGs and the whole mitogenomes.

**Figure 4:**
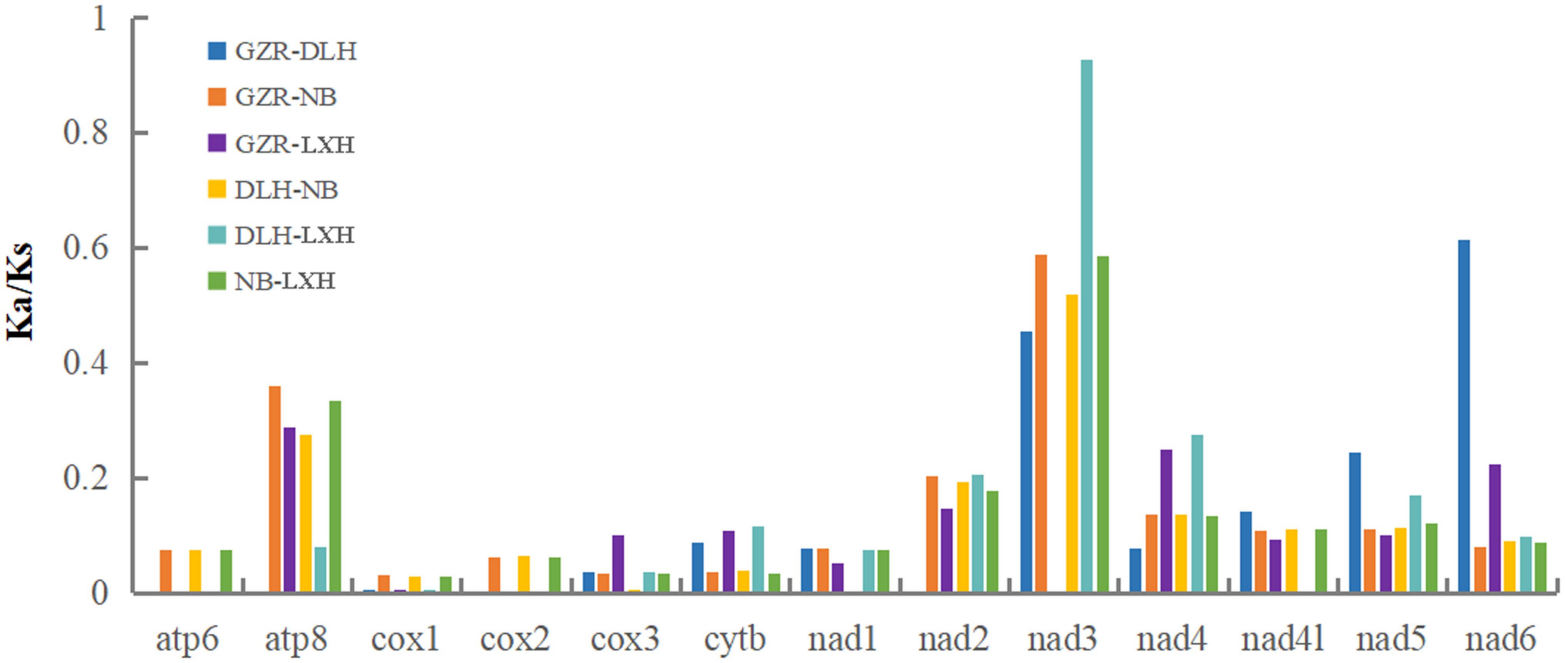
The evolutionary rate indicated by Ka/Ks for each PCG in the four representative mitogenomes of *P. tunguidus* (see Table 1). The names of 13 PCGs are shown on the *X*-axis, and the ratio of Ka/Ks is shown on the *Y*-axis.

To evaluate possible selection of mtDNA protein-coding sequences, Ka/Ks ratio was estimated for the four representative *P. tunguidus*. Their Ka/Ks ratio was less than 1 for all 13 PCGs (Figure 4), indicating that the PCGs were under purifying selection. COI had the lowest Ka/Ks ratio (0.001–0.03), i.e., this gene was under the strongest selective pressure. The ND3 gene had the highest Ka/Ks ratio (0–0.928).

To explore sequence divergence among the nine mitogenomes, we analyzed the pairwise genetic distances based on whole mitogenomes and 13 PCGs, respectively (Table 3). The whole mitochondrial genomes and PCGs of nine *P. tunguidus* showed significant genetic differentiation. For example, the genetic distance between the two *P. tunguidus* collected at NB and TB and those from the other seven sites is about 10% (mitogenomes K2P: 8.6–10.1%; PCGs K2P: 8.2–9.6%, Figure 3). The ATP8 gene had the smallest genetic distance among the 13 single PCGs (5.2–6.5%, Supplementary Table S5), whereas the ND4 gene had the largest genetic distance (12.8–13.4%, Supplementary Table S5). Additionally, the pair distance based on amino acid sequences (9.2–11.2%, Supplementary Table S6) was found to be higher than those based on nucleotides sequences. Mantel test showed no significant correlation between geographic distance and genetic differentiation (mitogenome, amino acid, PCGs, ribosomal RNA; Supplementary Figure S2).

**Table 3:** Pairwise genetic distance of 13 PCGs and whole genomes.

Samples	CBH	DLH	NB	TB	GZR	GZP	XK	ZWY	LXH
CBH		0.020	0.100	0.099	0.002	0.002	0.012	0.011	0.013
DLH	0.018		0.086	0.086	0.022	0.022	0.025	0.025	0.026
NB	0.095	0.082		0.001	0.100	0.100	0.101	0.101	0.101
TB	0.094	0.082	0.001		0.099	0.099	0.095	0.100	0.100
GZR	0.002	0.019	0.095	0.094		0.001	0.010	0.009	0.011
GZP	0.002	0.019	0.095	0.094	0.001		0.010	0.010	0.011
XK	0.011	0.023	0.096	0.095	0.009	0.009		0.002	0.002
ZWY	0.010	0.023	0.096	0.095	0.008	0.008	0.001		0.003
LXH	0.011	0.023	0.096	0.095	0.009	0.009	0.001	0.001	

Upper, whole genome genetic distance and Lower, protein-coding genes genetic distance.

#### Transfer and Ribosomal RNA Genes

**Figure 5:**
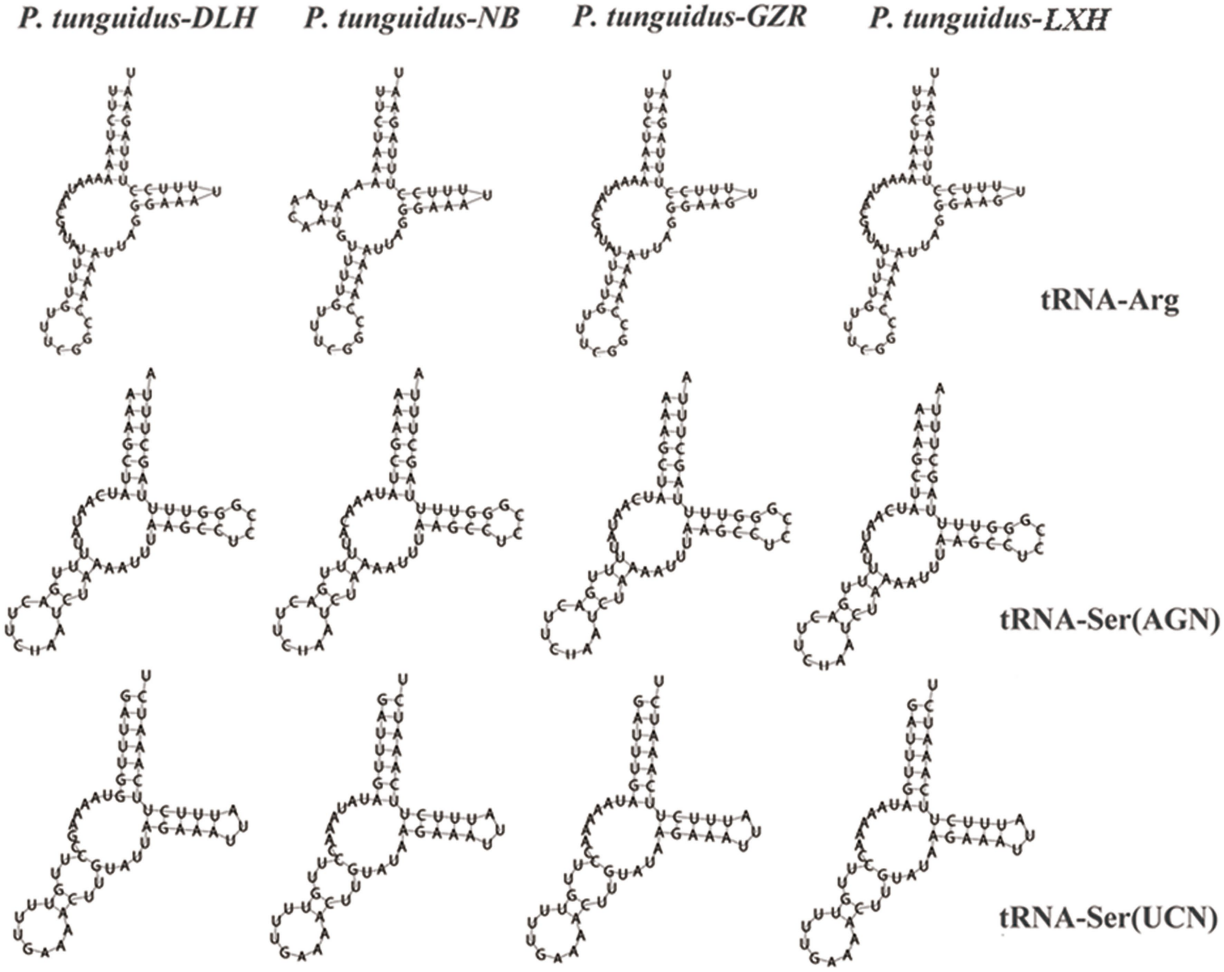
Secondary structures of three transfer tRNA genes from the four representative *P. tunguidus* mitogenome.

The length of tRNA genes ranged from 56 (tRNA-Ser and tRNA-Gly) to 68bp (tRNA-Trp). Most of the tRNAs were in a clover-leaf structure, except for tRNA-Arg, tRNA-Ser1 and tRNA-Ser2, which did not contain the entire dihydrouridine arm (Figure 5). However, tRNA-Arg of the two *P. tunguidus* from NB and TB in Guangxi Province had a complete clover-leaf structure. Mismatched base pairs G-U were found in most tRNAs.

The two rRNA genes were located between tRNA-Asp and tRNA-Phe, and isolated by tRNA-Trp (Table 1). The location and sequence feature of the rRNAs were identical to those observed in other Diaptomid species. However, the lengths of the 12S and 16S genes for the nine *P. tunguidus* were different from each other. The length of 12S was 641bp for the two *P. tunguidus* from TB and NB, and 643bp for those from the other seven sites. The length of 16S was 1,044bp for the *P. tunguidus* from TB and NB, 1043bp for that from DLH, and 1,040bp for those from ZWY, XK, and LXH, 1039bp for those from GZR, GZP, and CBH (Table 1). The rRNA genes in the *P. tunguidus* mitogenome exhibited a heavy AT nucleotide bias, with an A+T content of 76.78–77.48%. The skewed value of AT in the rRNAs of the nine *P. tunguidus* mitogenomes was from −0.015 to 0.005.

### Comparative Analysis of Our Mitochondrial Genomes With Those of Other Copepods

**Figure 6:**
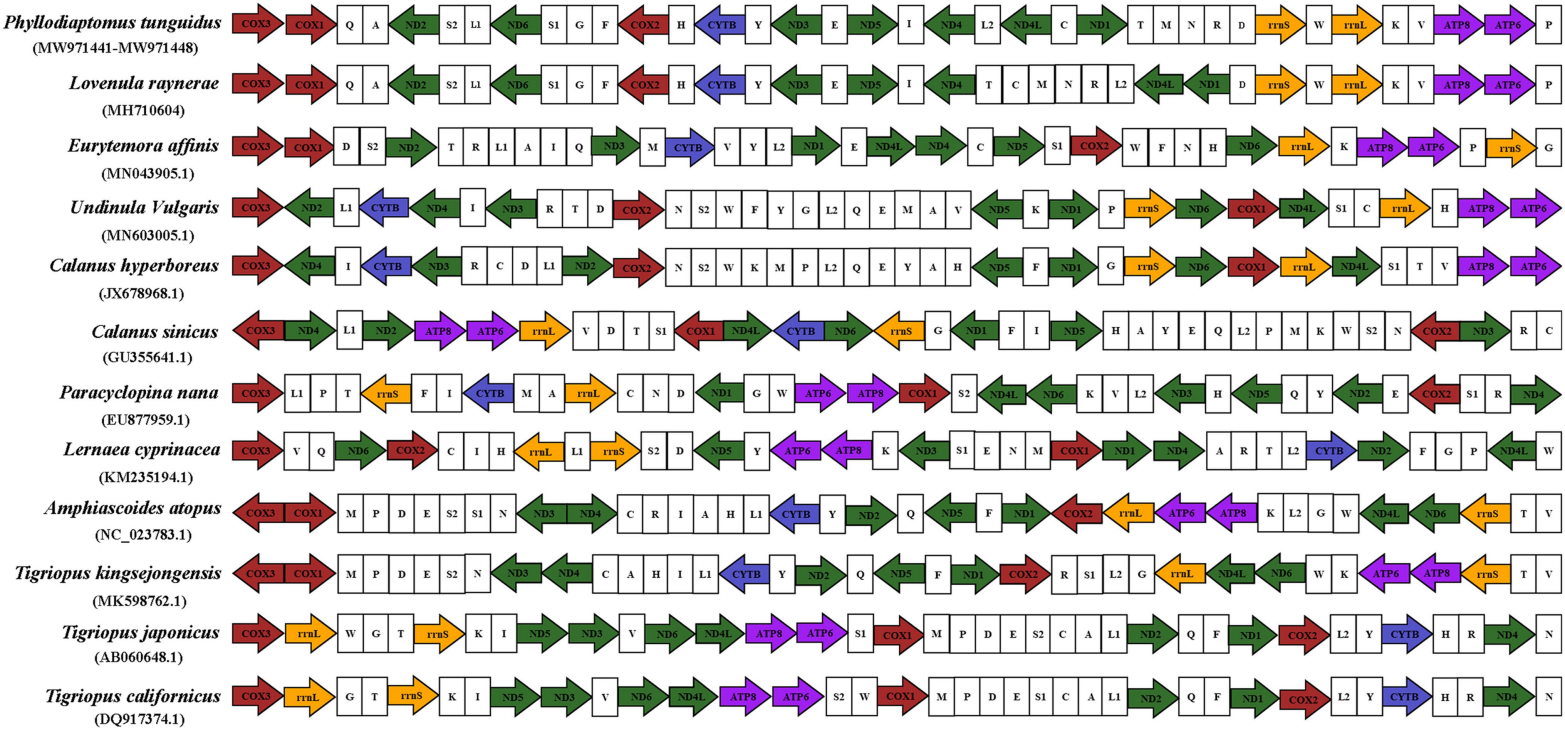
The mitochondrial genome composition and arrangement of Copepoda. The protein-coding genes (PCGs) are colored according to functional group and tRNA genes are portrayed using their single-letter amino acid code.

For comparison, we downloaded all available mitogenomes of Copepoda species in the NCBI database, with only two mitogenome available for Diaptomidae (Figure 6). A total of three orders and 11 species were examined for mitogenome structure, and PCGs and rRNA indicated the direction of the strands. Gene order in Copepoda was not conserved, and not only tRNA but also protein coding genes showed rearrangements. Our nine *P. tunguidus* had exactly the same gene order. *P. tunguidus* and *Lovenula raynerae*, both Diaptomidae, had the same PCGs gene order. The order of their six tRNAs between ND4 and ND1 was slightly different, but the order of the other tRNAs was consistent. There was a difference in PCGs and tRNA genes order between Diaptomidae (*P. tunguidus*) and Temoridae (*Eurytemora affinis*), Calanoidae (*Undinula vulgaris*, *Calanus hyperboreus*, and *Calanus sinicus*). Nevertheless, Diaptomidae genes were arranged in a relatively conservative order.

### Mitochondrial Phylogeny of *P. tunguidus*

**Figure 7:**
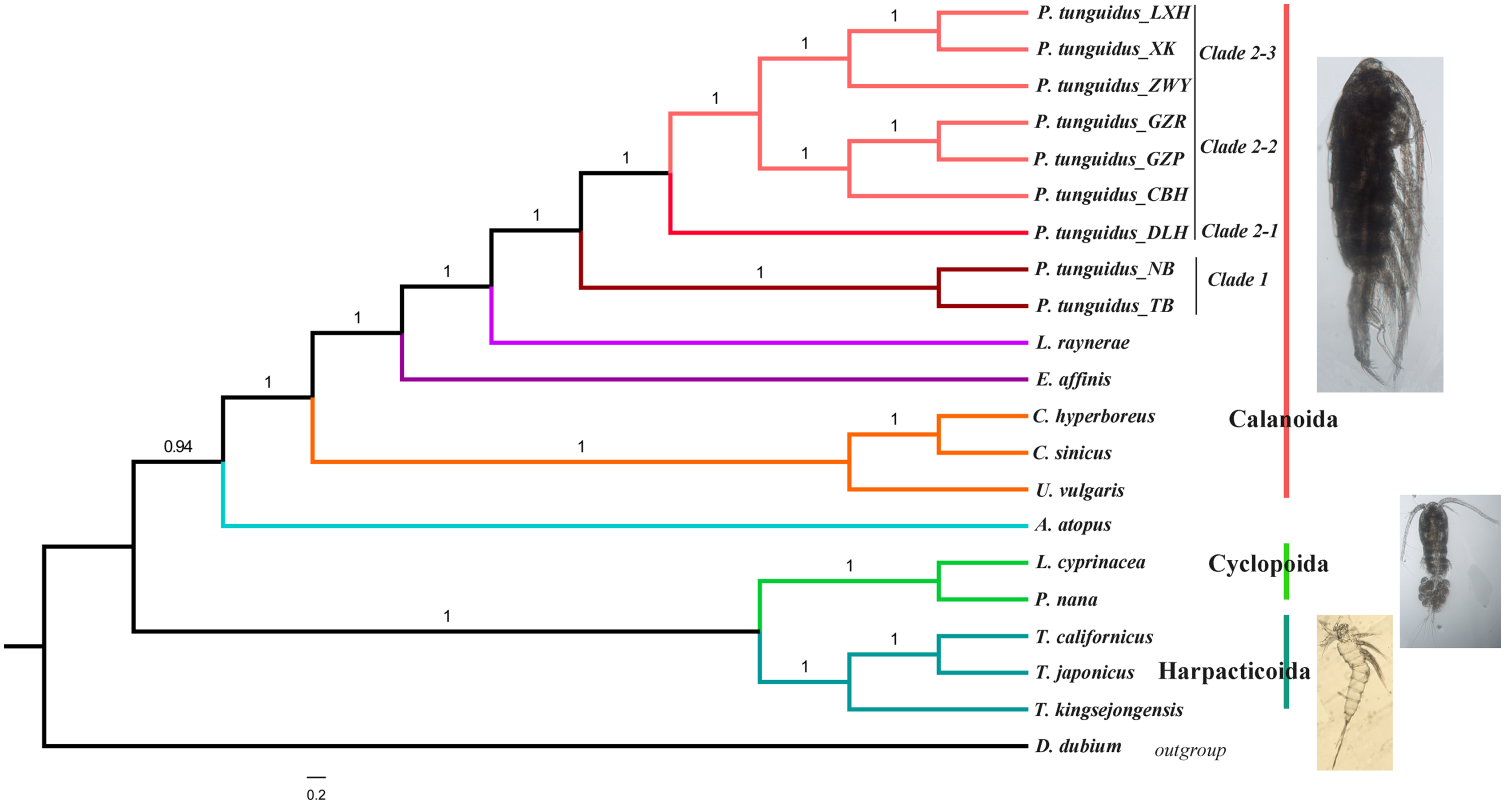
Phylogenetic relationship among Copepods reconstructed with 13 mitochondrial PCGs. Numbers at nodes represent the posterior probability in the BEAST analysis.

Although under the GTR+G+I substitution model, the Bayesian, NJ, and ML tree split all Copepoda into three orders (Calanoida, Cyclpoida, and Harpacticoida) with strong support (Figure 7). NJ, ML, and Bayesian analyses revealed a slightly different topology (Figure 7; Supplementary Figure S3). NJ and ML analyses showed that three orders, Calanoida, Cyclopoida, and Harpacticoida, form a monophyletic clade (Supplementary Figure S3). Harpacticoida is sister to Cyclopoida and Calanoida, Cyclopoida is recovered as sister group to Calanoida. Bayesian analyses showed that Calanoida and Cyclpoida form a monophyletic clade, but Harpacticoida not. *Amphiascoides atopus* of Harpacticoida is sister to Calanoida; Harpacticoida and Cyclpoida is the sister group of *A. atopus*. In addition, Bayesian analysis supported a sister relationship between Harpacticoida and Cyclopoida.

Phylogenetic analysis of the Calanoida showed that species of the Calanoidae occupy a basal position and species of Diaptomidae are at the apex of the evolutionary tree. Temoridae (*E. affinis*) was split from Calanoidae, whereas Diaptomidae (*L. laynerae* and *P. tunguidus* were clustered in one branch with high support, placing *P. tunguidus* in Diaptomidae) separated from Temoridae. NJ, ML, and Bayesian analyses based on mitochondrial genomes all split the nine *P. tunguidus* into two clades with high support (Clade 1 and 2, K2P=10%). The two *P. tunguidus* from TB and NB in Guangxi Province (near Vietnam) grouped in Clade 1, and the other seven *P. tunguidus* in Clade 2. Clade 1, the sister group of Clade 2 that contained Clade 2–1, Clade 2–2 and Clade 2–3, consisted of specimens from southern Guangxi Province. Clade 2–1, the sister of Clade 2–2 and Clade 2–3, occurred mainly in the center of Guangxi Province. Clade 2–2 and Clade 2–3 are in a sister group relationship, and they included the specimens from the upper and lower basins of Pearl River (Figure 1). Phylogeny of *P. tunguidus* in copepods using rrnL and rrnS was consistent with that based on PCGs (Supplementary Figure S4).

## Discussion

The mitogenomes of *P. tunguidus* were found to be similar to those of other copepods in terms of gene quantity and organizational structure (Ki et al., 2009; Jooste et al., 2019). Their characteristics conformed to the typical invertebrate mitogenome without gene insertion and deletion but with rearrangements. A common feature in metazoan mitochondrial genomes is bias toward a higher representation of nucleotides As and Ts leading to a subsequent bias in corresponding encoded amino acids (Wang et al., 2018; Ding et al., 2020; Liu et al., 2020; Sun et al., 2020). At this point, DNA with a high AT-content makes the double helix less stable because the AT pair is bound by two hydrogen bonds while GC pairs are bound by three such bonds (Yakovchuk et al., 2006); therefore, the high AT-content of mitogenome could have a higher mutation rate such that may accelerate the evolutionary process for Diaptomidae (Triant and DeWoody, 2006; Ding et al., 2020). Interestingly, the A+T content of PCGs at the 3rd codon position is higher than that of 1st and 2nd codon positions, and the G content at the 3rd codon position was lower than that 1st and 2nd codon positions. In addition, bias against T is prominent in the whole genome, all PCGs, the first, the second and the third codon of PCGs, especially at the second codon. A possible explanation is that the AT bias can selectively avoid the formation of stop codons and the loss of amino acids, and results in T-rich codons (Charneski et al., 2011; Ding et al., 2020). We determine preference for specific synonymous codons by evaluating the relative synonymous codon usage (RSCU; Granthan et al., 1980; Meganathan et al., 2012). The codon usage patterns were similar among the four *P. tunguidus*, with both 2- and 4-fold degenerate codons exhibiting over-usage of A and T at the third codon positions. The over-usage of A and T at the third codon is consistent with the observation in other animal species (Mindell et al., 1998; Ding et al., 2020; Sun et al., 2020). This might be related to genome bias, optimal selection of tRNA usage, or DNA repair efficiency (Crozier and Crozier, 1993).

The Ka/Ks ratio is an indicator of selective pressure acting on PCG (Yang and Bielawski, 2000; Hurst, 2002). It changed in the 13 PCGs, reflecting different functional constraints between genes (Muse, 2000). Specifically, COI had the lowest Ka/Ks ratio, indicating that this gene was under the strongest selective pressure. The ND3 gene had the highest Ka/Ks ratio, i.e., it was under the least selective pressure (Yang and Nielsen, 2000; Sun et al., 2011). Molecular evolution (Ka/Ks ratio) of single PCGs seems to be different in all nine *P. tunguidus*. However, it was consistent for the two *P. tunguidus* from NB and TB in Guangxi Province (near Vietnam). We failed to detect significant adaptive mutations in the nine mitogenomes. Therefore, purifying selection may be the predominant force governing mitogenome evolution in the species. Previous studies showed that energetic constraints are the main factors contributing to the evolution of mitochondrial-encoded proteins (Sun et al., 2011; Wagner et al., 2018). Combining the ratio of Ka/Ks and the genetic distance, we considered the *P. tunguidus* from NB and TB a unique lineage compared to the seven from the other sites, and the PCGs gene plays a crucial role in the evolutionary adaption under selection.

The ATP8 gene had the smallest genetic distance among the 13 single PCGs, whereas the ND4 gene had the largest distance, revealing different mutation pressures among genes (Sun et al., 2011). Additionally, the pair distance based on amino acid sequences was found to be higher than those based on nucleotides sequences. In other words, there are fewer synonymous substitutions than non-synonymous substitution in PCGs of the *P. tunguidus* mitogenomes, suggesting that some PCGs have undergone positive selection.

There is no evidence of resting eggs from *P. tunguidus* until now, thus it may be passively dispersed mainly following water flow. Isolation by distance (IBD) is expected in the dispersal limitation. However, the result showed no significantly correlation between the geographical distance and genetic differentiation (mitogenome, amino acid, PCGs, ribosomal RNA). This spatial pattern without IBD indicated that the current geographic distribution of *P. tunguidus* could have a complex historical origin. The geographical topography is indeed extremely complex in southern China (Clark et al., 2004; Xiang et al., 2007; Zheng, 2015), where Yunnan-Guizhou Plateau, Yunkai mountain and Shiwan mountain structure the landscape for *P. tunguidus*. Genetic differentiation between adjacent populations such as GZR and GZP, LXH and ZWY indicated that there are strong environmental filters or selections.

The exact position of 22 tRNA genes of the eight *P. tunguidus* was identified as being the same genomic position observed for our published *P. tunguidus* mitogenome (Zhang et al., 2020b). The tRNA gene in all the nine *P. tunguidus* had anti-codons that match the invertebrate mitochondrial code (Weydmann et al., 2017). However, tRNA-Arg of the two *P. tunguidus* from NB and TB in Guangxi Province had the complete clover-leaf structure. The structure of tRNA-Ser1 was not the typical clover-leaf structure that occurs in other invertebrates and vertebrates (Wolstenholme, 1992; Hong et al., 2008; Yang et al., 2019). The secondary structure of lacking a dihydrouridine arm is likely related its structural compensation mechanism among tRNA arms (Steinberg and Cedergren, 1994). Mismatched and wobble pairs are commonly found in invertebrate tRNAs and can be corrected by post-transcriptional RNA editing processes (Lavrov et al., 2000). Two rRNA genes were identified for *P. tunguidus* through alignment with other Diaptomidae mitogenomes (Jooste et al., 2019; Zhang et al., 2020b). The rRNA genes exhibited a heavy AT nucleotide bias in the *P. tunguidus* mitogenomes. The RNA-Seq analysis in many species demonstrates that a TAA stop codon is created by post-transcriptional polyadenylation that changes T or TA residues to a complete TAA stop codon. Therefore, the rRNA with a high AT-content species has a less stable double helix, which helps to implement the above mechanisms (Yakovchuk et al., 2006).

Phylogenetic analysis indicates that Copepoda species fall into three orders. Calanoida and Cyclpoida for a monophyletic clade, but Harpacticoida do not. Calanoida is identified as the most recently diverging order of the three, and Diaptomidae is the most recently diverging family of the Calanoida. Morphological taxonomic studies are generally consistent the current molecular phylogenies except for the *A. atopus* in Harpacticoida.

In this study, we clarified the phylogeny and hidden diversity of *P. tunguidus* in southern China. Focusing on the mitochondrial genomes of nine *P. tunguidus*, the phylogeny showed that the two *P. tunguidus* from NB and TB (Clade 1) are separated from the other seven. A phylogenetic tree based on mitogenomes and *Cytb* showed that Clade 1 is restricted to the southern Guangxi Province. In addition, two *P. tunguidus* populations (TB and NB) near Vietnam can be considered older, if we look at the fact that most species in *Phyllodiaptomus* are limited to Southeast Asia (Dumont and Reddy, 1993, 1994; Dumont et al., 1996; Sanoamuang and Yindee, 2001; Sanoamuang and Teeramaethee, 2006; Sanoamuang and Watiroyram, 2020). In contrast, Clade 2 is widely distributed in many relatively isolated basins, including the Lancnagjiang River (Yunnan Dali), Upper Pearl River (Yunnan, Kunming), Lower Pearl River (Gaozhou, Guangzhou and Shaoguan), the Minjiang River (Fujian, Fuzhou) and the Yangtze River (Hunan, Changsha; Supplementary Table S2 and Supplementary Figure S1). The spatial proximity among these basins allows *P. tunguidus* populations in the Pearl River basin to expand into nearby basins easily, especially in flooding seasons. At the same time, the hypothesis of active river capture in southwestern China may explain the close relationship of populations between the Yangtze and Upper Pearl River (Clark et al., 2004; Zheng, 2015). In addition to the two strongly supported mitogenome Clades, a high level of mtDNA genetic differentiation, observed between the two Clades (5.2–13.4%) supports a deep divergence among *P. tunguidus* populations. This finding is shared with several small-sized freshwater fish species in China, e.g., *Hemiculter leucisculus* (Chen et al., 2017), *Opsariichthys bidens* (Perdices et al., 2005), *Rhynchocypris oxycephalus* (Yu et al., 2014).

Similar to the phylogenetic relationship, the gene arrangement of mitochondrial genomes demonstrates that Calanoida have a more complete classification system, while the classification of Harpacticoida is currently unresolved.

## Conclusion

The nine mitogenomes of *P. tunguidus* have similar size and gene arrangement, but their base composition, genetic distance and tRNA structure indicate a large genetic differentiation between mitogenomes. Deep genetic differentiation occurs between two clades of widely different geographic origins. This differentiation is also shown in the difference in the structures of tRNA and rRNA. Two clades have reached the level of subspecies or cryptic species. The mitogenome-based phylogeny of Copepoda further shows that Diaptomidae is the most recently diverging family of Calanoida and that *P. tunguidus* is at the evolutionary apex of the family.

## Data Availability Statement

The original contributions presented in the study are included in the article/Supplementary Material; further inquiries can be directed to the corresponding author.

## Author Contributions

B-PH: conceptualization. X-LZ, PL, and S-LX: methodology and software. X-LZ: formal analysis and writing – original draft preparation. X-LZ, QZ, and PL: data curation. X-LZ, B-PH, ER, and HD: writing – review and editing. All authors have read and agreed to the published version of the manuscript.

## Conflict of Interest

The authors declare that the research was conducted in the absence of any commercial or financial relationships that could be construed as a potential conflict of interest.

## Publisher’s Note

All claims expressed in this article are solely those of the authors and do not necessarily represent those of their affiliated organizations, or those of the publisher, the editors and the reviewers. Any product that may be evaluated in this article, or claim that may be made by its manufacturer, is not guaranteed or endorsed by the publisher.

## Footnotes

^1^ http://www.ncbi.nlm.nih.gov/Blast.cgi

## Abbreviations

Mitogenome: Mitochondrial genome
rRNA: Ribosomal RNA
tRNA: Transfer RNA
RSCU: Relative synonymous codon usage
PCG: Protein-coding gene
NJ: Neighbor-Joining
ML: Maximum-likelihood
AICc: Akaike information criterion
MCMC: Monte Carlo Markov Chain
