# Supplementary material for: Geographic Variation of Phyllodiaptomus tunguidus Mitogenomes: Genetic Differentiation and Phylogeny
Source: Front Genet. 2021 Aug 31;12:711992. doi: 10.3389/fgene.2021.711992 (PMC8439380; doi:10.3389/fgene.2021.711992)
Supplement: Supplementary file 1 [file Data_Sheet_1.doc]

Supplementary Material

**Table S1** List of species used to construct the phylogenetic tree.

| **Order** | **Family** | **species** | **GenBank number** |
| --- | --- | --- | --- |
| Calanoida | Diaptomidae | *Lovenula raynerae* | MH710604 |
| Calanoida | Diaptomidae | *Phyllodiaptomus tunguidus* | MN927223 |
| Calanoida | Diaptomidae | *Phyllodiaptomus tunguidus* | **MW971441** |
| Calanoida | Diaptomidae | *Phyllodiaptomus tunguidus* | **MW971442** |
| Calanoida | Diaptomidae | *Phyllodiaptomus tunguidus* | **MW971443** |
| Calanoida | Diaptomidae | *Phyllodiaptomus tunguidus* | **MW971444** |
| Calanoida | Diaptomidae | *Phyllodiaptomus tunguidus* | **MW971445** |
| Calanoida | Diaptomidae | *Phyllodiaptomus tunguidus* | **MW971446** |
| Calanoida | Diaptomidae | *Phyllodiaptomus tunguidus* | **MW971447** |
| Calanoida | Diaptomidae | *Phyllodiaptomus tunguidus* | **MW971448** |
| Calanoida | Temoridae | *Eurytemora affinis* | MN043905 |
| Calanoida | Calanoidae | *Undinula vulgaris* | MN603005 |
| Calanoida | Calanoidae | *Calanus hyperboreus* | JX678968 |
| Calanoida | Calanoidae | *Calanus sinicus* | GU355641 |
| Cyclopoida | Cyclopettidae | *Paracyclopina nana* | EU877959 |
| Cyclopoida | Lernaeidae | *Lernaea cyprinace* | KM235194 |
| Harpacticoida | Miraciidae | *Amphiascoides atopus* | NC023783 |
| Harpacticoida | Harpacticidae | *Tigriopus californicus* | DQ917374 |
| Harpacticoida | Harpacticidae | *Tigriopus japonicus* | AB060648 |
| Harpacticoida | Harpacticidae | *Tigriopus kingsejongensis* | MK598762 |

**Table S2** The geographical information for *P. tunguidus* samples which used to construct the *Cytb* phylogenetic tree.

| **Code** | **Population** | **Geographical coordinates** | **Altitude** |
| --- | --- | --- | --- |
| YNDL | Dali, Yunnan | 99.57/26.08 | 2070 |
| YNKM | Kunming, Yunnan | 102.83/24.77 | 1992 |
| GZGY | Guizhou, Guiyang | 106.69/26.60 | 1121 |
| GXGL | Guilin, Guangxi | 110.09/25.30 | 222 |
| GXNN | Nanning, Guangxi | 108.24/22.87 | 93 |
| GXCZ | Chongzuo, Guangxi | 107.65/22.37 | 127 |
| GDSG | Shaoguan, Guangdong | 113.29/24.76 | 79 |
| GDLXH | Guangzhou, Gunagdong | 113.46/23.76 | 192 |
| GDGZ | Maoming, Guangdong | 110.88/21.68 | 80 |
| GDJY | Jieyang, Guangdong | 116.08/23.05 | 36 |
| FJFZ | Fujian,Fuzhou | 119.31/26.11 | 84 |
| HNCS | Changsha, Hunan | 113.00/28.21 | 63 |

**Table S3** Annotation of the other five complete mitochondrial genomes of *P. tunguidus.*

| **Gene** | **CBH**  **GZP** |  | **TB** |  | **XK**  **ZWY** |  | **Size** | **Strand** | **Codon** |  | **Anticodon** |
| --- | --- | --- | --- | --- | --- | --- | --- | --- | --- | --- | --- |
|  | From | To | From | To | From | To |  |  | start | Stop |  |
| tRNA ^Ala^ | 1 | 60 | 1 | 60 | 1 | 60 | 60 | L |  |  | tgc/tgc/tgc |
| ND2 | 59 | 1027 | 59 | 1027 | 59 | 1027 | 969 | L | ATA/ATA/ATA | TAG/TAG/TAG |  |
| tRNA^Ser^ | 1028 | 1083 | 1028 | 1083 | 1028 | 1083 | 56 | L |  |  | tga/tga/tga |
| tRNA ^Leu^ | 1082 | 1143 | 1082 | 1143 | 1082 | 1143 | 62 | L |  |  | gtc/gtc/gtc |
| ND6 | 1142 | 1603 | 1141 | 1605 | 1142 | 1603 | 462/465/462 | L | ATT/ATT/ATT | TAA/TAA/TAA |  |
| tRNA ^Ser^ | 1603 | 1660 | 1603 | 1660 | 1603 | 1660 | 58 | L |  |  | tct/tct/tct |
| tRNA ^Pro^ | 1660 | 1721 | 1660 | 1721 | 1660 | 1721 | 62 | L |  |  | tcc/tcc/tcc |
| tRNA^Lys^ | 1723 | 1783 | 1723 | 1783 | 1723 | 1783 | 61 | L |  |  | gaa/gaa/gaa |
| COX2 | 1783 | 2484 | 1783 | 2484 | 1783 | 2484 | 702 | L | ATT/ATT/ATT | TAA/TAA/TAA |  |
| tRNA ^Val^ | 2503 | 2562 | 2503 | 2562 | 2503 | 2562 | 60 | L |  |  | gtg/gtg/gtg |
| CYTB | 2562 | 3695 | 2562 | 3695 | 2562 | 3695 | 1134 | L | ATG/ATG/ATG | TAA/TAA/TAA |  |
| tRNA ^Met^ | 3698 | 3759 | 3698 | 3759 | 3698 | 3759 | 62 | L |  |  | gta/gta/gta |
| ND3 | 3767 | 4120 | 3767 | 4120 | 3767 | 4120 | 354/356/  354 | L | ATT/ATG/ATT | TAA/TAA/TAA |  |
| tRNA ^Glu^ | 4162 | 4226 | 4160 | 4223 | 4162 | 4226 | 65 | H |  |  | ttc/ttc/ttc |
| ND5 | 4227 | 5912 | 4224 | 5909 | 4227 | 5912 | 1686 | H | ATA/ATA/ATA | TAA/TAA/TAA |  |
| tRNA ^Ile^ | 5911 | 5972 | 5908 | 5969 | 5911 | 5972 | 62 | H |  |  | gat/gat/gat |
| ND4 | 5970 | 7250 | 5967 | 7247 | 5970 | 7250 | 1281 | L | ATA/ATA/ATA | TAA/TAA/TAA |  |
| tRNA^Leu^ | 7260 | 7323 | 7257 | 7320 | 7260 | 7323 | 64 | L |  |  | taa/taa/taa |
| ND4L | 7319 | 7648 | 7316 | 7645 | 7319 | 7648 | 330 | L | ATT/ATT/ATT | TAA/TAA/TAA |  |
| tRNA ^Thr^ | 7686 | 7746 | 7684 | 7744 | 7686 | 7746 | 61 | H |  |  | gca/gca/gca |
| ND1 | 7791 | 8711 | 7790 | 8710 | 7791 | 8711 | 921 | H | ATA/ATA/ATA | TAG/TAG/TAG |  |
| tRNA ^Cys^ | 8711 | 8771 | 8710 | 8770 | 8711 | 8771 | 61 | H |  |  | tgt/tgt/tgt |
| tRNA ^Tyr^ | 8772 | 8833 | 8771 | 8832 | 8772 | 8833 | 62 | H |  |  | cat/cat/cat |
| tRNA ^Asn^ | 8836 | 8895 | 8835 | 8895 | 8836 | 8895 | 60/61/60 | H |  |  | gtt/gtt/gtt |
| tRNA ^Arg^ | 8899 | 8956 | 8898 | 8955 | 8899 | 8956 | 58 | H |  |  | tcg/tcg/tcg |
| tRNA ^Asp^ | 8957 | 9019 | 8956 | 9018 | 8957 | 9019 | 63 | H |  |  | tag/tag/tag |
| rrnS | 9018 | 9660 | 9017 | 9657 | 9018 | 9660 | 643/641/  643 | H |  |  |  |
| tRNA ^Trp^ | 9666 | 9733 | 9663 | 9730 | 9666 | 9733 | 68 | H |  |  | tca/tca/tca |
| rrnL | 9734 | 10772 | 9730 | 10773 | 9734 | 10773 | 1039/1044/1040 | H |  |  |  |
| tRNA ^Phe^ | 10772 | 10831 | 10773 | 10832 | 10773 | 10832 | 60 | H |  |  | ttt/ttt/ttt |
| tRNA ^His^ | 10830 | 10891 | 10831 | 10892 | 10831 | 10892 | 62 | H |  |  | tac/tac/tac |
| ATP8 | 10892 | 11053 | 10893 | 11054 | 10893 | 11054 | 162 | H | ATT/ATT/ATT | TAA/TAA/TAA |  |
| ATP6 | 11056 | 11757 | 11057 | 11758 | 11057 | 11758 | 702 | H | ATG/ATG/ATG | TAG/TAA/TAG |  |
| tRNA ^Gly^ | 11761 | 11816 | 11762 | 11817 | 11759 | 11822 | 56/56/64 | H |  |  | tgg/tgg/tgg |
| COX3 | 11818 | 12609 | 11819 | 12610 | 11819 | 12610 | 792 | H | ATG/ATG/ATG | TAA/TAA/TAA |  |
| COX1 | 12629 | 14164 | 12631 | 14166 | 12630 | 14165 | 1536 | H | ATT/ATT/ATT | TAA/TAA/TAA |  |
| tRNA^Gln^ | 14165 | 14230 | 14167 | 14232 | 14166 | 14231 | 66 | H |  |  | ttg/ttg/ttg |
| D-Loop | 14231 | - | 14233 | - | 14232 | - | - |  |  |  |  |

**Table S4** A+T contents, AT-skew of the four representative mitogenomes of *P. tunguidus.*

|  | **Location** | **Whole genome** | **Protein-coding genes** | **1^st^ codon position** | **2^nd^ codon position** | **3^rd^ codon position** | **rRNAs** |
| --- | --- | --- | --- | --- | --- | --- | --- |
| A + T Contents | GZP | 69.4 | 66.9 | 62.7 | 63.9 | 73.8 | 77.1 |
|  | CBH | 69.4 | 66.8 | 62.7 | 63.9 | 73.9 | 77.1 |
|  | TB | 68.8 | 66.4 | 62.4 | 64.0 | 72.8 | 76.6 |
|  | XK | 69.2 | 66.8 | 62.7 | 64.1 | 73.8 | 77.3 |
|  | ZWY | 69.2 | 66.8 | 62.7 | 64.1 | 73.8 | 77.2 |
| AT-skew | GZP | -0.004 | -0.227 | -0.107 | -0.47 | -0.118 | 0.007 |
|  | CBH | -0.003 | -0.226 | -0.107 | -0.47 | -0.119 | 0.008 |
|  | TB | -0.009 | -0.236 | -0.111 | -0.47 | -0.134 | -0.011 |
|  | XK | -0.004 | -0.228 | -0.107 | -0.47 | -0.122 | 0.007 |
|  | ZWY | -0.0028 | -0.228 | -0.107 | -0.47 | -0.122 | 0.006 |

**Table S5** Pairwise genetic distance for ND4 and ATP8

| **Location** | **GZR** | **DLH** | **GZP** | **NB** | **TB** | **XK** | **CBH** | **ZWY** | **LXH** |
| --- | --- | --- | --- | --- | --- | --- | --- | --- | --- |
| GZR |  | 0.006 | 0.001 | 0.052 | 0.052 | 0.013 | 0.001 | 0.013 | 0.013 |
| DLH | 0.004 |  | 0.006 | 0.059 | 0.059 | 0.006 | 0.006 | 0.006 | 0.006 |
| GZP | 0.001 | 0.004 |  | 0.052 | 0.052 | 0.013 | 0.001 | 0.013 | 0.013 |
| NB | 0.130 | 0.128 | 0.130 |  | 0.001 | 0.065 | 0.052 | 0.065 | 0.065 |
| TB | 0.131 | 0.129 | 0.131 | 0.001 |  | 0.065 | 0.052 | 0.065 | 0.065 |
| XK | 0.012 | 0.009 | 0.012 | 0.133 | 0.134 |  | 0.013 | 0.001 | 0.001 |
| CBH | 0.002 | 0.002 | 0.002 | 0.129 | 0.13 | 0.012 |  | 0.013 | 0.013 |
| ZWY | 0.012 | 0.009 | 0.012 | 0.133 | 0.134 | 0.001 | 0.012 |  | 0.001 |
| LXH | 0.013 | 0.010 | 0.013 | 0.132 | 0.133 | 0.001 | 0.013 | 0.001 |  |

**Table S6** Pairwise genetic distance for amino acid and rRNA.

| **Location** | **GZR** | **DLH** | **GZP** | **NB** | **TB** | **XK** | **CBH** | **ZWY** | **LXH** |
| --- | --- | --- | --- | --- | --- | --- | --- | --- | --- |
| GZR |  | 0.01 | 0.001 | 0.052 | 0.052 | 0.004 | 0.01 | 0.003 | 0.004 |
| DLH | 0.019 |  | 0.01 | 0.042 | 0.041 | 0.012 | 0.010 | 0.012 | 0.012 |
| GZP | 0.001 | 0.019 |  | 0.052 | 0.052 | 0.004 | 0.001 | 0.003 | 0.004 |
| NB | 0.109 | 0.092 | 0.109 |  | 0.001 | 0.051 | 0.053 | 0.052 | 0.051 |
| TB | 0.109 | 0.092 | 0.109 | 0.001 |  | 0.050 | 0.052 | 0.051 | 0.050 |
| XK | 0.010 | 0.024 | 0.010 | 0.111 | 0.111 |  | 0.004 | 0.001 | 0.001 |
| CBH | 0.003 | 0.018 | 0.003 | 0.109 | 0.109 | 0.013 |  | 0.004 | 0.004 |
| ZWY | 0.010 | 0.024 | 0.010 | 0.111 | 0.111 | 0.001 | 0.012 |  | 0.001 |
| LXH | 0.011 | 0.024 | 0.011 | 0.111 | 0.111 | 0.001 | 0.013 | 0.001 |  |


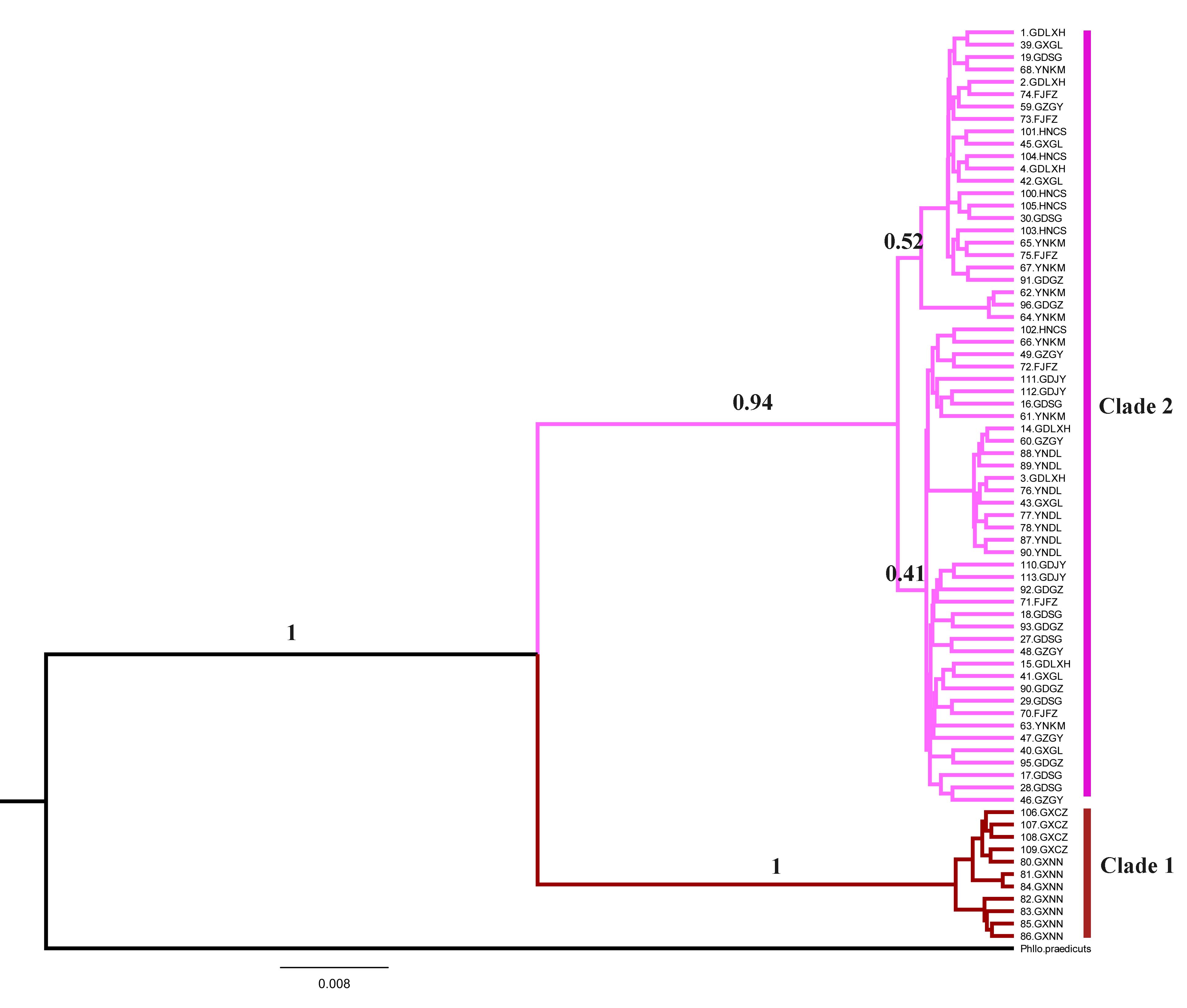


**Figure S1**. Bayesian phylogenetic tree of *Cytb* fragments for *Phyllodiaptomus tunguidus*. *P. praedictus* was used as the outgroup.


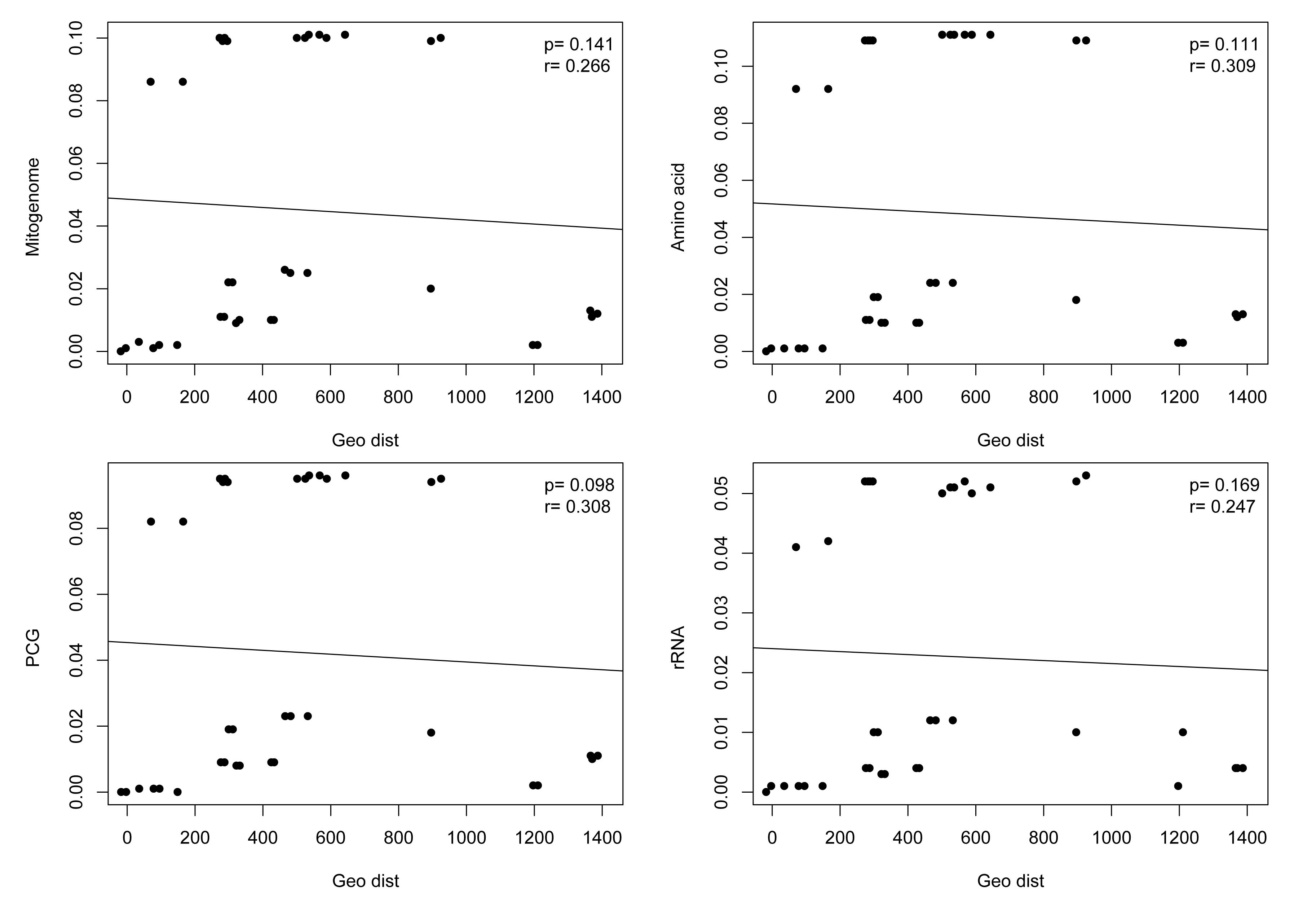


**Figure S2**. Mantel test of genetic differentiation (mitogenome, amino acid, protein-coding genes, ribosomal RNA) with geographic distance (distance between two sampling sites).


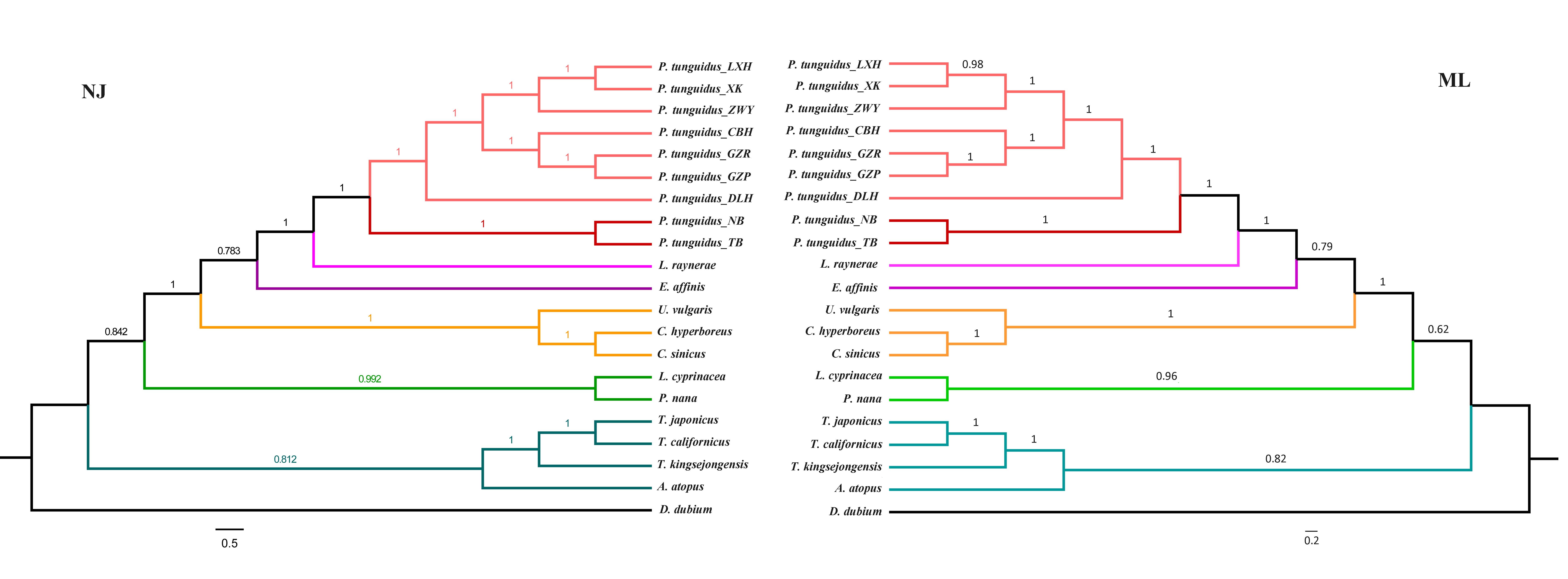


**Figure S3.** Phylogenetic relationship among Copepods based on 13 mitochondrial PCGs. Numbers at nodes represent the posterior probability for the ML and NJ analyses.


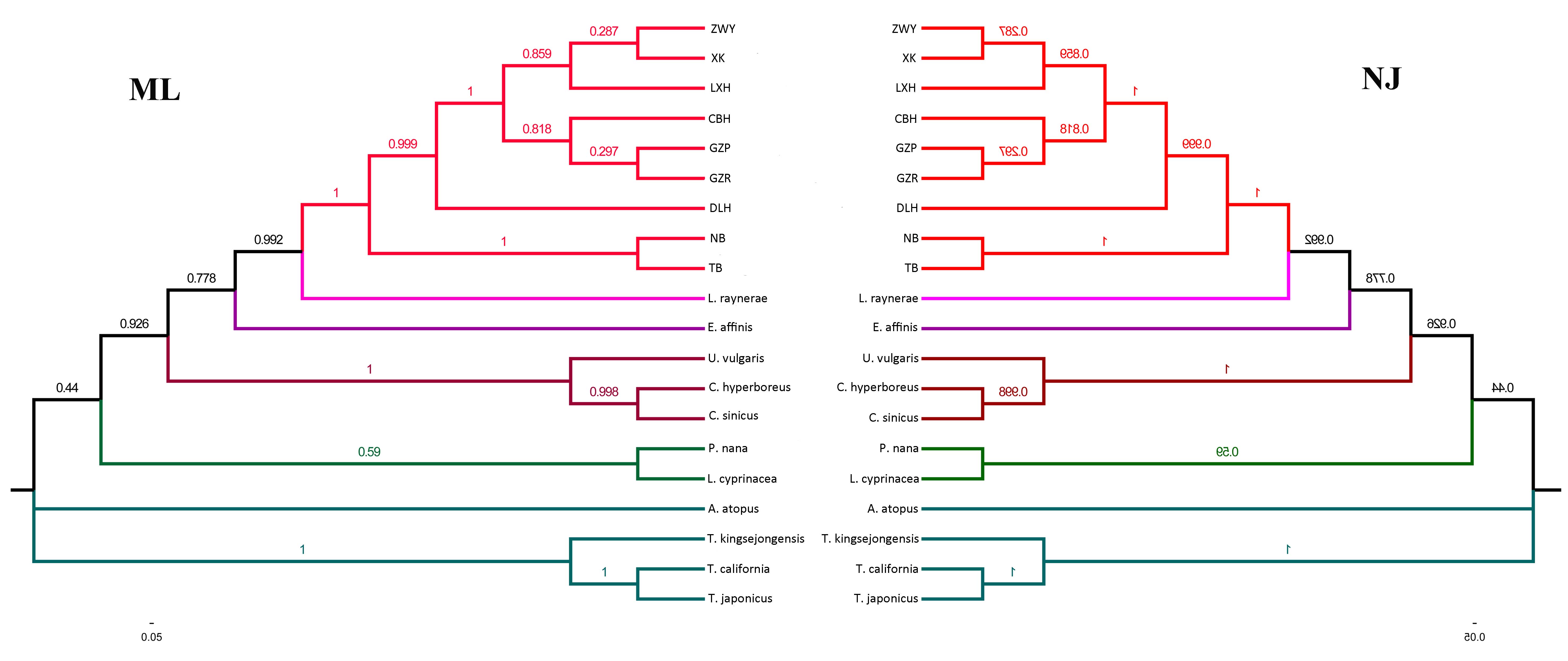


**Figure S4.** Phylogenetic relationship among Copepods based on rRNA. Numbers at nodes represent the posterior probability for the ML and NJ analyses.
